# H3K4me3-related lncRNAs signature and comprehensive analysis of H3K4me3 regulating tumor immunity in lung adenocarcinoma

**DOI:** 10.1186/s12931-023-02418-1

**Published:** 2023-05-03

**Authors:** Tao Fan, Mingchuang Zhu, Shan Muhammad, Chu Xiao, Shuofeng Li, He Tian, Yu Liu, Liyan Xue, Bo Zheng, Chunxiang Li, Jie He

**Affiliations:** 1 Department of Thoracic Surgery, National Clinical Research Center for Cancer/Cancer Hospital, National Cancer Center, Chinese Academy of Medical Sciences and Peking Union Medical College, Beijing, 100021 China; 2 Department of Colorectal Surgery, National Clinical Research Center for Cancer/Cancer Hospital, National Cancer Center, Chinese Academy of Medical Sciences and Peking Union Medical College, Beijing, 100021 China; 3 Department of Pathology, National Clinical Research Center for Cancer/Cancer Hospital, National Cancer Center, Chinese Academy of Medical Sciences and Peking Union Medical College, Beijing, 100021 China

**Keywords:** H3K4me3 modification, LncRNA, Tumor immunity, LUAD

## Abstract

**Backgroud:**

The role of epigenetic modifications in tumorigenesis has been widely reported. However, the role and mechanism of H3K4me3 modification in lung adenocarcinoma (LUAD) are rarely reported systematically. We, therefore, sought to analyze the characteristics of LUAD associated with H3K4me3 modification, build an H3K4me3-lncRNAs score model to predict the prognosis of patients with LUAD and clarify the potential value of H3K4me3 in immunotherapy of LUAD.

**Methods:**

We evaluated H3K4me3-lncRNA patterns and H3K4me3-lncRNA scores of 477 LUAD samples based on 53 lncRNAs closely correlated to H3K4me3 regulators and comprehensive analyzed the role of these patterns in tumorigenesis and tumor immunity. Using Gene set variation analysis (GSVA), we systematically evaluated the H3K4me3 level of every sample and deeply analyzed the effect of H3K4me3 on the prognosis of LUAD. In addition, we included two independent immunotherapy cohorts to study the impact of high H3K4me3 score on the prognosis of patients. We also used an independent cohort with 52 matched paraffin specimens of LUAD to verify the impact of high H3K3me3 expression on the prognosis of patients.

**Results:**

We identified three H3K4me3-lncRNA patterns that exhibited specific immune characteristics. Characterized by immunosuppressive and increased TGFβ-mediated epithelial-mesenchymal transition (EMT), patients with high H3K4me3-lncRNA score had a poor overall survival and decreased H3K4me3 score. H3K4me3 score was significantly positively correlated with CD4^+^T-cell and CD8^+^T-cell activation, programmed cell death and immune checkpoints (ICs) expression, and was negatively correlated with MYC pathway, TP53 pathway, and cell proliferation. Patients with high H3K4me3 score showed elevated expression of ICs, potentiated CD4 T-cell and CD8 T-cell activation, increased programmed cell death, and suppressed cell proliferation and TGFβ-mediated EMT. Patients with high H3K4me3 score and high expression of CTLA4, ICOS, TIGIT, PDCD1LG2, IDO1, CD274, PDCD1, LAG3, or HAVCR2 had the best survival advantage. Two independent immunotherapy cohorts verified that patients with high H3K4me3 score showed an increased inflamed tumor microenvironment (TME) phenotype and enhanced anti-PD-1/L1 immunotherapy response. Immunohistochemistry (IHC) data from 52 matched paraffin specimens of LUAD confirmed that the protein level of H3K4me3 in tumor was significantly lower than that of paracancerous tissues and H3K4me3 brought significant survival benefits to patients with LUAD.

**Conclusions:**

We build an H3K4me3-lncRNAs score model to predict the prognosis of patients with LUAD. More importantly, this study revealed characteristics of H3K4me3 modification in LUAD and clarified the important potential role of H3K4me3 on tumor immunotherapy and patients’ survival.

**Supplementary Information:**

The online version contains supplementary material available at 10.1186/s12931-023-02418-1.

## Background

Abnormal activity of gene promoter region is a key initiating factor for tumorigenesis. CpG dinucleotides are unevenly distributed in the human genome. They are characteristically distributed in the promoter region and exon regions to regulate gene expression [1]. Trimethylation of lysine 4 on histone H3 protein subunit (H3K4me3) is a special methylation marker found on active genes [2, 3], and the levels of H3K4me3 at CpG island largely determine gene transcription [4]. Although the enrichment of H3K4me3 in the gene promoter indicates the high expression of this gene, the effect of H3K4me3 on tumors is still controversial. LY6K-AS abrogation repressed malignant characteristics of A549 cells, which was attributed to the process that the knockdown of LY6K-AS significantly reduced the H3K4me3 marker at the promoter [5]. Similarly, it was reported that A549 cells with stable KDM6A knockdown showed decreased H3K4me3 and anti-tumor phenotype [6]. Above studies indicated that high H3K4me3 of histone was a factor that promoted the progression of LUAD. Nonetheless, some studies showed that high H3K4me3 was beneficial to the overall survival of cancer patients. Heward and his colleagues found that KDM5-inhibition elevated H3K4me3 levels and elicited an anti-proliferative response [7]. In the study of pancreatic cancer, scholars also found that the inhibition of KMT2D facilitated tumor growth and led to the loss of H3K4me3 marker [8]. Therefore, as one of the essential parts of epigenetic modification, the role and mechanism of H3K4me3 in LUAD still need to be further explored.

H3K4me3 was usually directly regulated by methyltransferases (“writers”), demethylases (“erasers”) and binding proteins (“readers”). The well-known methyltransferases regulating H3K4me3 contained KMT2A/2B/2 C/2D, SETD2, SETD7, et al. [9], while the demethylases included KDM1A/2A/2B/4A/5A/5B/5 C/5D /7A [10]. Additionally, the “readers”, such as SPIN1, CXXC1, C17orf49, PHF23, ZCWPW1, BRD4, SPP1, PHF13, ZMYND8, ING1/2/4, and PHF7 could regulate H3K4me3 by recognizing and binding its motif [11]. All these regulators were finely regulated by other molecules in the cell while regulating H3K4me3 modification. Long non-coding RNA (LncRNA) is a type of key small molecule involved in cell bioinformatics that has been widely reported in recent years. LncRNA-PM maintains cerebellar synaptic integrity via Pax6/Mll1-mediated H3K4me3 [12]. Lnc_3712 inhibited nuclear reprogramming by KDM5B-mediated H3K4me3 [13]. Although these studies have reported in detail that lncRNA can interfere with cell signaling pathways by directly or indirectly regulating H3K4me3, molecular regulation in organisms is extremely complicated. There has been no comprehensive analysis of the regulatory effects of lncRNA and H3K4me3 from the transcriptomics level.

The infiltration of immune cells and the activation of immune pathways determine tumor immunotherapy’s effectiveness. The discovery of ICs and the clinical application of their inhibitors has been a significant breakthrough in tumor immunotherapy in recent years. Although more than ten kinds of ICs (PDCD1, PDCDLG2, LAG3, IDO1, et al.) have been discovered, the clinical efficacy of tumor immunotherapy still needs to be improved. Recent studies have shown that epigenetic modifications are essential in tumor immunity [14–17]. It was reported that four kinds of epigenetic drugs (modulators of DNA methylation, histone acetylation, histone methylation, and BET readers of lysine acetylation) were used in the treatment of tumors [18]. Through appropriate intervention epigenetic modification, these drugs can regulate the activity of CD4^+^ and CD8^+^ T lymphocytes and NK cells to exert anti-tumor effects [18].

In this study, we constructed three distinct H3K4me3-lncRNA clusters for the first time based on the expression of H3K4me3 regulators and their closely related lncRNAs. Furthermore, we demonstrated the specific TME immune characteristics of each cluster. The high consistency between H3K4me3-lncRNA score and immune characteristics revealed that H3K4me3 played an essential role in remodeling the tumor immune microenvironment. For that, we evaluated the H3K4me3 level of each sample and further analyzed the potential molecular mechanisms of H3K4me3 regulating tumor progression. This research confirmed the anti-cancer effect and immunological mechanism of H3K4me3 modification in LUAD with a large sample size, which help us deeply understand the immune regulation of TME and provided a new insights for studying the role of H3K4me3 modification in oncogenesis and tumor immunogenicity.

## Methods

### Data source and preprocessing

The flowchart of this study was shown in Fig. S1. Public gene-expression data and clinical annotation were searched in The Cancer Genome Atlas (TCGA) database and Gene-Expression Omnibus (GEO). TCGA-LUAD transcriptome data for 551 samples used in the article was downloaded from the Genomic Data Commons (GDC, https://portal.gdc.cancer.gov/). A total of 54 normal sample data and 477 (20 duplicates have been eliminated) tumor sample data were included in the analysis. The Fragments Per Kilobase of exon model per Million mapped fragments (FPKM) values were converted to transcripts per kilobase million (TPM) values. IMvigor210, a clinical trials, was publicly obtained. Locally advanced or metastatic urothelial cancer patients who were treatment-naive and ineligible for cisplatin-containing chemotherapy or patients who have disease progression during or after the previous platinum-based chemotherapy regimen received atezolizumab (anti-PD-L1 anti-body) treatment (1,200 mg i.v. every 3 weeks) in IMvigor210 cohort [19, 20]. All gene expression data of IMvigor210 and detailed clinical annotations were downloaded from http://research-pub.gene.com/IMvigor210 Core Biologies. Another immunotherapeutic cohort GSE91061 in which melanoma patients received Nivolumab treatment (anti-PD-1 anti-body) was included to verify the important role of H3K4me3 in immune-checkpoint blockage [21]. The GSE91061 dataset from PRJNA356761 cohort was downloaded from National Library of Medicine (https://www.ncbi.nlm.nih.gov/) and handled with a method of quantile normalization.

### H3K4me3 regulators and annotation of lncRNAs

According to the literature, we collected 29 H3K4me3 regulators containing 7 writers (KMT2A, KMT2B, KMT2C, KMT2D, SETD2, SETD7, EZH2), 9 erasers (KDM1A, KDM2A, KDM2B, KDM4A, KDM5A, KDM5B, KDM5C, KDM5D, KDM7A), and 13 readers (SPIN1, CXXC1, C17orf49, PHF23, ZCWPW1, BRD4, SPP1, PHF13, ZMYND8, ING1, ING2, ING4, PHF7). The standard of |Pearson R|>0.6 and *p* < 0.001 were used to screen out 331 lncRNAs that were closely related to H3K4me3. Univariate Cox regression analysis was performed to identify 53 prognostic factors (*p* < 0.05).

### Unsupervised cluster generation and identification of differentially expressed genes (DEGs)

Using R package of “ConsensusClusterPlus”, 477 patients were divided into three patterns based on the expression of identified 53 lncRNAs. This classification’s stability and reliability of were guaranteed by a 1000-time cycle computation [22]. The R package of “limma” was used to identify DEGs between different clusters with a significance criteria of |logFC|>0.2 and adjusted *p* value < 0.05.

### Biological pathway analysis

To evaluate the biological pathway involved in H3K4me3-lncRNA clusters and compare the difference in biological process between different H3K4me3-lncRNA score types, we performed gene ontology (GO), kyoto encyclopedia of genes and genomes (KEGG), gene set enrichment analysis (GSEA), gene set variation analysis (GSVA) enrichment analysis using R packages of “org.Hs.eg.db”, “clusterProfiler”, “fgsea”, “GSVA”, “limma”. The gene sets of “h.all.v7.4.symbols.gmt” and “c5.go.bp.v7.4.symbols.gmt” were downloaded from MSigDB database and used to perform GSEA_HALLMARK term and GSEA_GO term analysis respectively. FDR < 0.05 was set as the cutoff value for functional annotation.

### H3K4me3-lncRNA score generation and survival analysis

In order to better display the specific H3K4me3-lncRNA characteristics of the individual patient, we performed a principal component analysis (PCA), which was widely used to score samples [16, 23–25], and the signature score was generated from PC 1 and 2. All Kaplan-Meier curves in this research were generated by R packages of “survminer” and “survival”. A *p*-value < 0.05 meant that there was a significant difference in overall survival (OS) between the groups.

### Collection of human tissues

Fifty-two formalin-fixed, paraffin-embedded LUAD and paired normal tissue samples were collected from the National Cancer Center, Cancer Hospital, Chinese Academy of Medical Sciences (Beijing, China). All the 52 patients were pathologically diagnosed and underwent radical resection from January to December 2012.

### Immunohistochemistry (IHC) and evaluation

Three µm thick parafin sections of tumor were deparaffinized, dewaxed and rehydrated. The slides were stained with primary rabbit antibody and secondary antibody sequentially. Anti-human H3K4me3 (CST, #9751, 1:2000) was used in this study. The staining intensity was divided into four levels: “-” (negative), “+” (low), “++” (moderate) or “+++” (high), represented by 0, 0.1, 0.2, 0.3 points respectively. Positive cell frequency score were evaluated as 0 (< 5%), 1 (5%<25%), 2 (25-50%), 3(50-75%), or 4(> 75%). The final IHC score = staining intensity point × Positive cell frequency score. The 52 patients was divided into H3K4me3 high group and H3K4me3 low group by optimal cutoff value of all the final IHC score.

### Statistical analysis

All the statistical analyses were conducted using R version 3.6.1. The prognostic value of H3K4me3-lncRNA score and other biomarkers were evaluated by R package of “time ROC”. The area under the curve (AUC) reflected the predictive power. Univariate Cox and multivariate Cox regression were performed to verify that high H3K4me3-lncRNA score was an independent risk factor. Each subgroup was determined by the optimal cutoff value based on “surv-cutpoint” function of survminer R package. The Kaplan-Meier method evaluated OS scores in every subgroup, and a log-rank test calculated the difference between subgroups. A comparison of three or more groups was made with one-way ANOVA. The chi-square test and student’s *t*-test were used to evaluate the differences between categorical and continuous variables respectively. Bilateral *p* value and *p*-value < 0.05 were criteria for meaningful results.

## Results

### Expression characteristics of lncRNA closely related to H3K4me3 in LUAD

To identify the lncRNAs closely related to H3K4me3, 29 H3K4me3 regulators were included. A correlation analysis between lncRNAs and H3K4me3 regulators was used to extracted 568 H3K4me3-related lncRNAs (|Pearson R|>0.6 and *p* < 0.001). The network of H3K4me3 regulators and 568 lncRNAs is shown in Fig. S2A and Table S1. A further univariate Cox regression analysis was performed to identify 53 prognostic factors (Fig. S2B, Table S2). Finally, the comparison of these lncRNAs in tumor and normal tissues was shown in Fig. S2C.

### Unsupervised clustering of 53 H3K4me3-related lncRNAs and biological features of each pattern

**Fig. 1:**
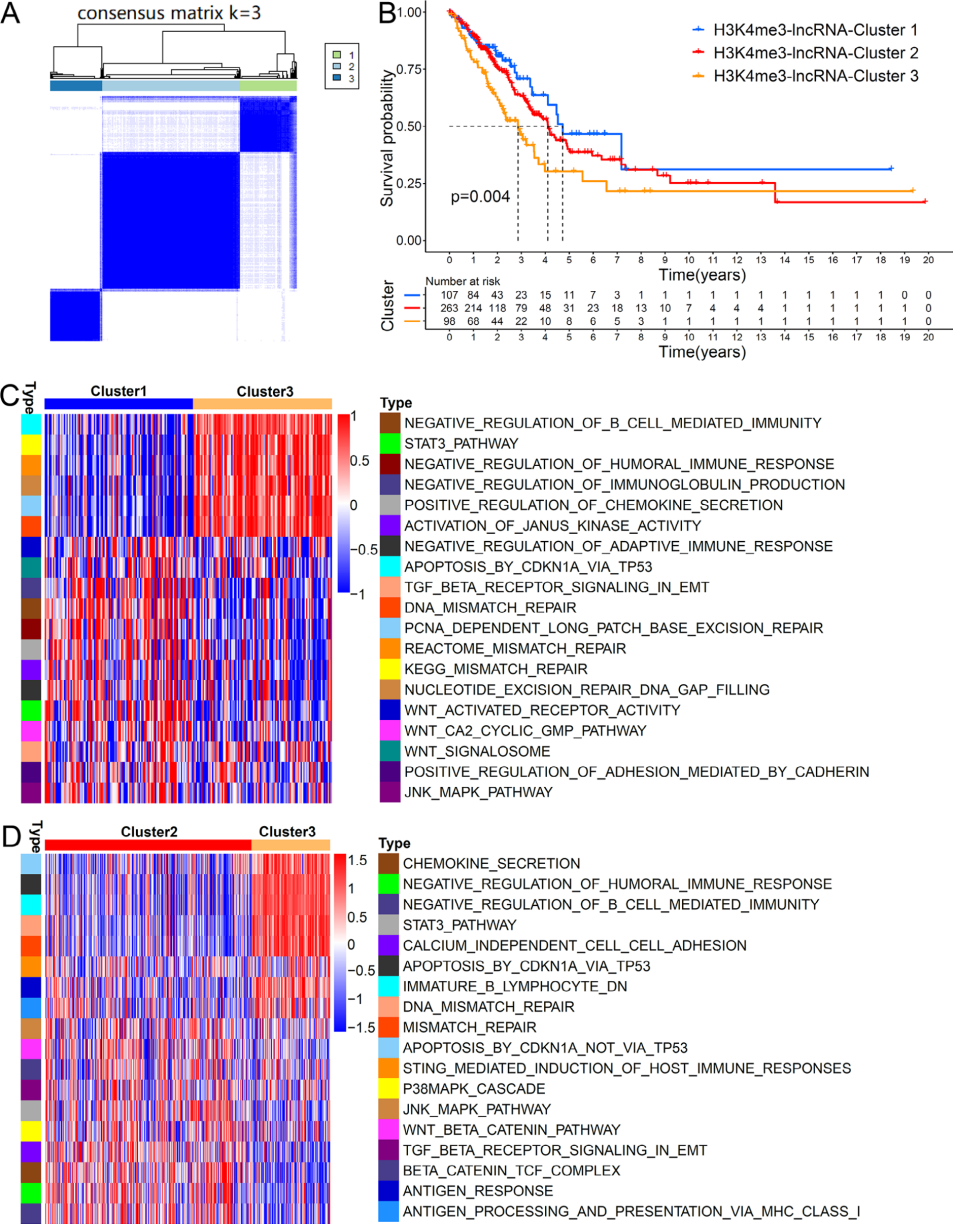
Unsupervised clustering of 53 H3K4me3-related lncRNAs and biological features of each pattern using GSVA analysis. (A) Consensus clustering matrices for k = 3 based on 53 H3K4me3-related lncRNAs. (B) Kaplan–Meier curves showing the predictive value of the three H3K4me3-related lncRNA patterns. (C) (D) GSVA enrichment analysis for the distinct H3K4me3-related lncRNA patterns. The activation states of biological processes were visualized by heatmap. Red and blue represented activated and repressed pathways respectively. C, H3K4me3-lncRNA-Cluster 1 vs. H3K4me3-lncRNA-Cluster 3; D, H3K4me3-lncRNA-Cluster 2 vs. H3K4me3-lncRNA-Cluster 3

Using R package of “ConsensusClusterPlus”, 477 patients were divided into three patterns based on optimal consensus matrix k value (k = 3) (Fig. 1A). Survival analysis for the three identified H3K4me3-lncRNA patterns indicated that cluster 1 had a clear survival advantage, and cluster 3 showed poor long-term survival (Fig. 1B). GSVA analysis revealed that, compared with cluster 1, cluster 3 was mainly involved in mismatched repair, WNT pathway activation and immunosuppression (Fig. 1C). Similarly, cluster 3 showed obvious immunosuppression and tumorigenesis compared with cluster 2 (Fig. 1D).

**Fig. 2:**
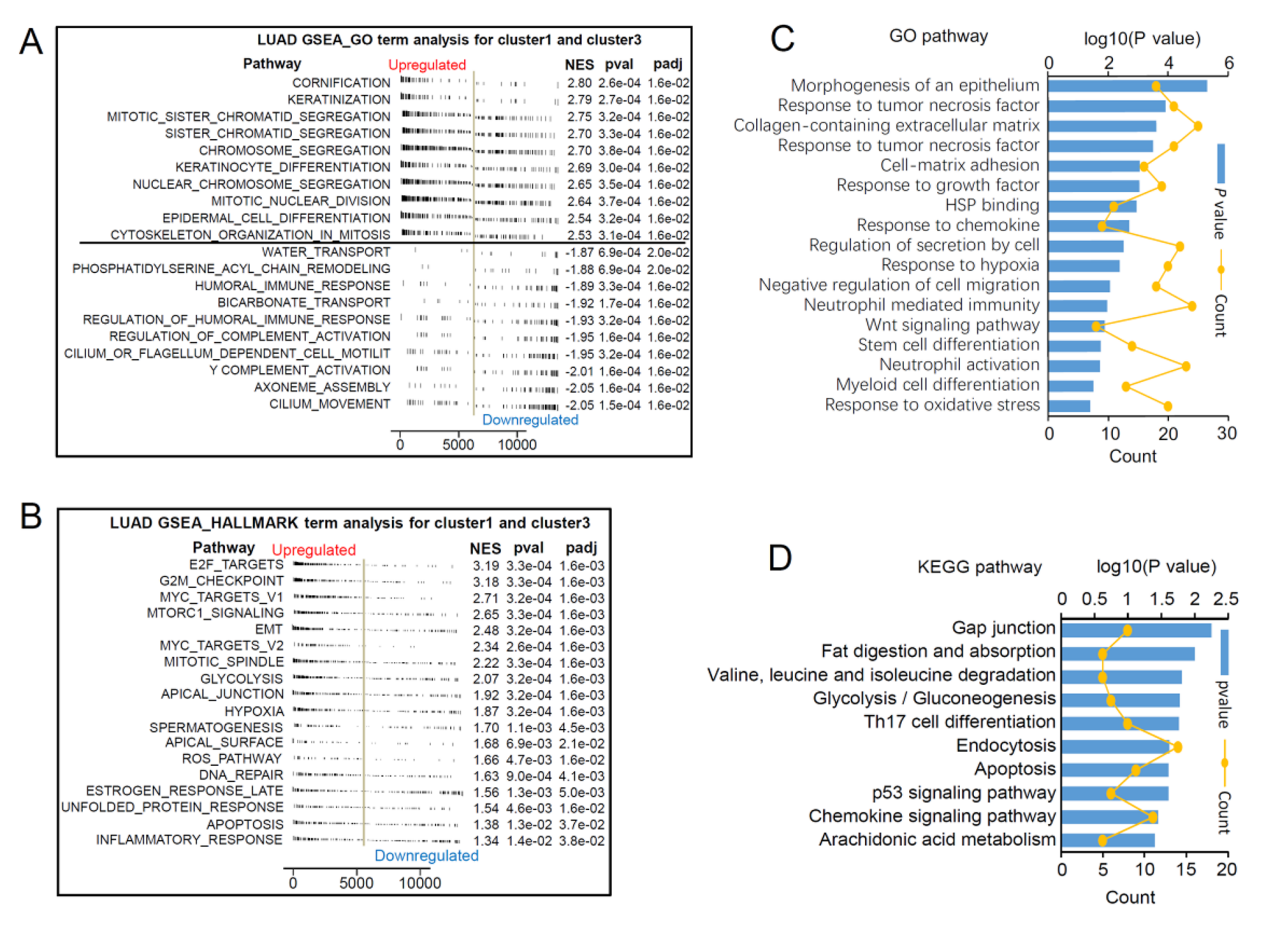
The potential molecular biological mechanisms of the identified three patterns. (A) GSEA_GO term analysis for H3K4me3-lncRNA-Cluster 1 and H3K4me3-lncRNA-Cluster 3 (adj. *p* < 0.05; *p* value < 0.05; NES, enrichment score; The top 10 positive and negative pathways selected by NES were provided). (B) GSEA_HALLMARK term analysis for H3K4me3-lncRNA-Cluster 1 and H3K4me3-lncRNA-Cluster 3. (adj. *p* < 0.05; *p* value < 0.05; NES, enrichment score). (C) GO and KEGG (D) pathways significantly associated with tumor progress based on the 592 differently expressed genes among the three patterns

Considering the significant difference in survival between cluster 1 and cluster 3, we further performed GSEA analysis for these two patterns. As shown in Fig. 2A, compared with cluster 1, in cluster 3, top 10 upregulated pathways were all involved in cell proliferation and differentiation. In comparison, ten downregulated pathways were mainly immune response and complement activation. A GSEA_HALLMARK term analysis found that epithelial-mesenchymal transition (EMT), MYC, mTOR, and cell division were significantly activated in H3K4me3-lncRNA cluster 3 (Fig. 2B). To give insight into the biological behavior of each H3K4me3 modification pattern, we take the intersection between every two H3K4me3-lncRNA clusters, and 592 DEGs (adjusted *p* < 0.05) were identified (Table S3). GO (Fig. 2C) and KEGG (Fig. 2D) analysis for the DEGs revealed that H3K4me3 modification was closely related to immunity, morphogenesis of an epithelium, WNT signal pathway and cell adhesion.

### The molecular characteristics of H3K4me3-lncRNA score and its predictive value in prognosis of LUAD

**Fig. 3:**
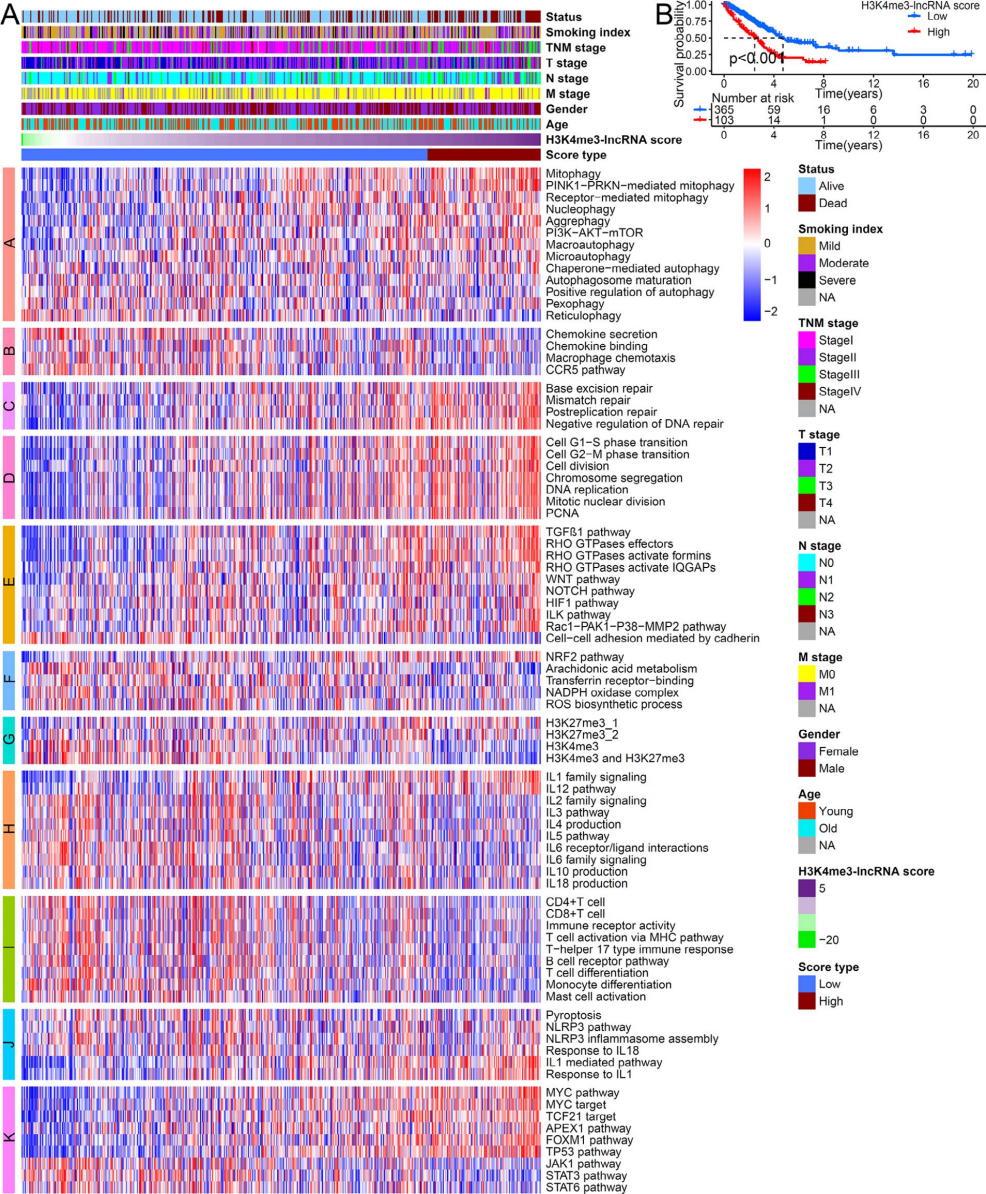
The molecular characteristics of H3K4me3-lncRNA score and its impact on survival. (A) Heat map showing the relationship between H3K4me3-lncRNA score and core biological pathways. Columns of the heat map represented clinical features grouped by H3K4me3-lncRNA score type. Rows of the heat map showed pathway enrichment score grouped by the following special biological process: A: autophagy signature, B: chemokine pathways signature, C: cell repair signature, D: cell cycle signature, E: epithelial-mesenchymal transition (EMT) signature, F: ferroptosis signature, G: histone trimethylation modification signature, H: IL pathways signature, I: immune cell activation pathways signature, J: pyroptosis signature, K: other tumor-related pathways signature. (B) Survival analysis for high and low H3K4me3-lncRNA score patterns based on 477 patients with LUAD

Considering the individual heterogeneity of LUAD patients and the complexity of H3K4me3 modification, we scored each patient based on the expression of 53 H3K4me3-related lncRNAs. We first performed univariate and multivariate Cox regression analysis based on H3K4me3- lncRNAs score and clinical features. The result verified that high H3K4me3-lncRNAs score was an independent risk factor for poor prognosis of LUAD (Table S4). Then, to clarify its mechanism, we studied the relationship between H3K4me3-lncRNA score and immune activation, cell cycle, programmed cell death, cell repair, MYC-mediated pathways, TP53-mediated pathways, STAT3-mediated pathways, FOXM1-mediated pathways, ILs-mediated pathways, and chemokines-mediated pathways. Fig. 3A showed distribution signature of the H3K4me3-lncRNA score, the patient’s clinical characteristics, and biological signaling pathways. Grouping patients based on the optimal cut-off value, patients with low H3K4me3-lncRNA score showed a significant survival advantage (Fig. 3B). We take intersection between high and low H3K4me3-lncRNA score group (|logFC|>1 and adjusted *p* value < 0.05), and 177 DEGs were identified (Table S5). GO and KEGG analysis for the 177 DEGs indicated that epidermis development, epidermal cell differentiation, chemokine receptor binding, cytokine-cytokine receptor, IL and immune response were closely related to H3K4me3-lncRNA score (Fig. S3A, S3B). A GSEA_HALLMARK term analysis found that EMT, apical junction, P53 pathway, MYC, mTOR, and cell cycle were significantly enriched in high-H3K4me3-lncRNA score group (Fig. S3C). Another GSEA_GO term analysis verified that patients with high H3K4me3-lncRNA scores had increased epidermal and epithelial cell differentiation, and decreased B cell-mediated immune response (Fig. S3D). All these results fully demonstrated the molecular mechanism of poor prognosis in patients with high H3K4me3-lncRNA score.

### The impact of H3K4me3-lncRNA score on tumor immunity

**Fig. 4:**
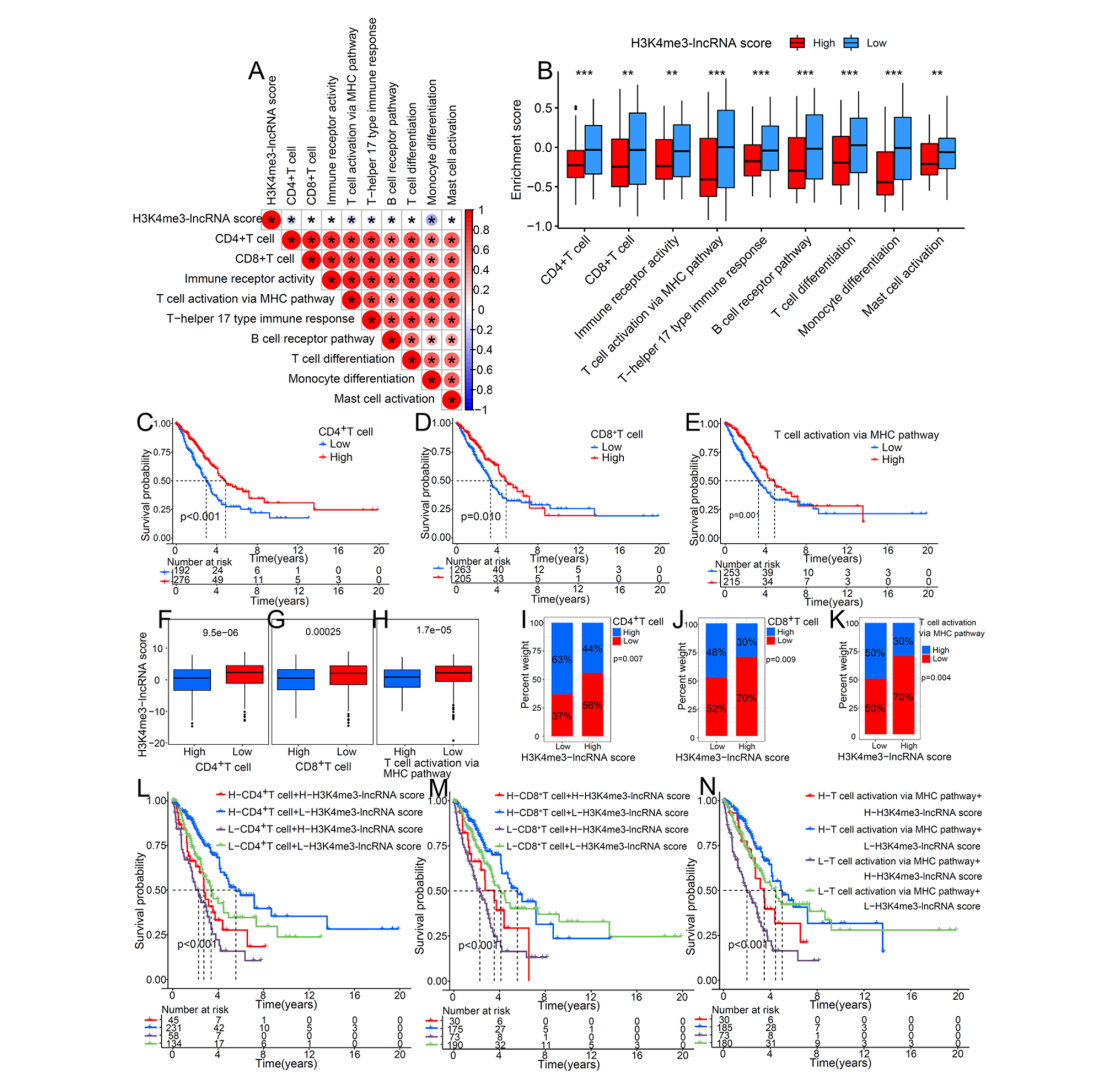
The relationship between H3K4me3-lncRNA score and immune cell activation-related pathway. (A) Correlation analysis between H3K4me3-lncRNA score and immune cell activation-related pathway. (B) Comparison of immune cell activation-related pathway enrichment score between high and low H3K4me3-lncRNA score groups. Enrichment score was calculated by GSVA analysis. (C) (D) (E) Survival analyses showed a significant impact of immune cell activation related molecular subtypes on outcome. (F) (G) (H) Differences in H3K4me3-lncRNA score between high-and low- CD4^+^ T cell, CD8^+^ T cell, and T cell activation via MHC pathway score groups. (I) (J) (K) The proportion of different score groups of indicated molecular subtypes in the two H3K4me3-lncRNA score patterns. (L) Kaplan–Meier curves showing the predictive value of subgroups stratified by H3K4me3-lncRNA score and CD4^+^ T cell activation. (M) Survival analysis for subgroup patients classified by H3K4me3-lncRNA score and CD8^+^ T cell activation. (N) Kaplan-Meier curves showing the predictive value of subgroups stratified by H3K4me3-lncRNA score and T cell activation via MHC pathway. (*p < 0.05; **p < 0.01; ***p < 0.001)

Next, we focused on the regulation of the H3K4me3-lncRNA score and tumor immunity. As showed in Fig. 4A, H3K4me3-lncRNA score was strongly negatively correlated with T cell- and B cell-mediated anti-tumor immune signaling pathways, and the immune pathways activation in patients with high H3K4me3-lncRNA score was significantly lower than that in patients with low H3K4me3-lncRNA score (Fig. 4B). Immune pathway activation status was indicated by the enrichment score which GSVA calculated. The results revealed that patients with high activation of CD4^+^T cell (Fig. 4C), CD8^+^T cell (Fig. 4D), or T cell activation via MHC pathway (Fig. 4E) had obviously survival advantages. As expected, patients with high activation of CD4^+^T cell (Fig. 4F), CD8^+^T cell (Fig. 4G), or T cell activation via MHC pathway (Fig. 4H) had lower H3K4me3-lncRNA score. A Chi-square test revealed that there was a smaller proportion of patients with activated CD4^+^T cell (Fig. 4I), CD8^+^T cell (Fig. 4J), or T cell activation via MHC pathway(Fig. 4K) in the high-H3K4me3-lncRNA score populations. The above results fully confirmed that H3K4me3 modification was closely related to CD4^+^T-and CD8^+^T-mediated tumor immunity. The T cell activation status was the intrinsic molecular biological basis of the H3K4me3-lncRNA score for predicting patient prognosis. Fig. 4 L showed that patients with low- H3K4me3-lncRNA score and high-CD4^+^T enrichment score had the best survival advantage. Similar results were obtained in Fig. 4M and N. These survival analysis results showed that this H3K4me3-lncRNA score could provide better patient enrollment criteria for tumor immunotherapy targeting CD4^+^ or CD8^+^ T cells.

### The effect and mechanism of H3K4me3 modification on LUAD

**Fig. 5:**
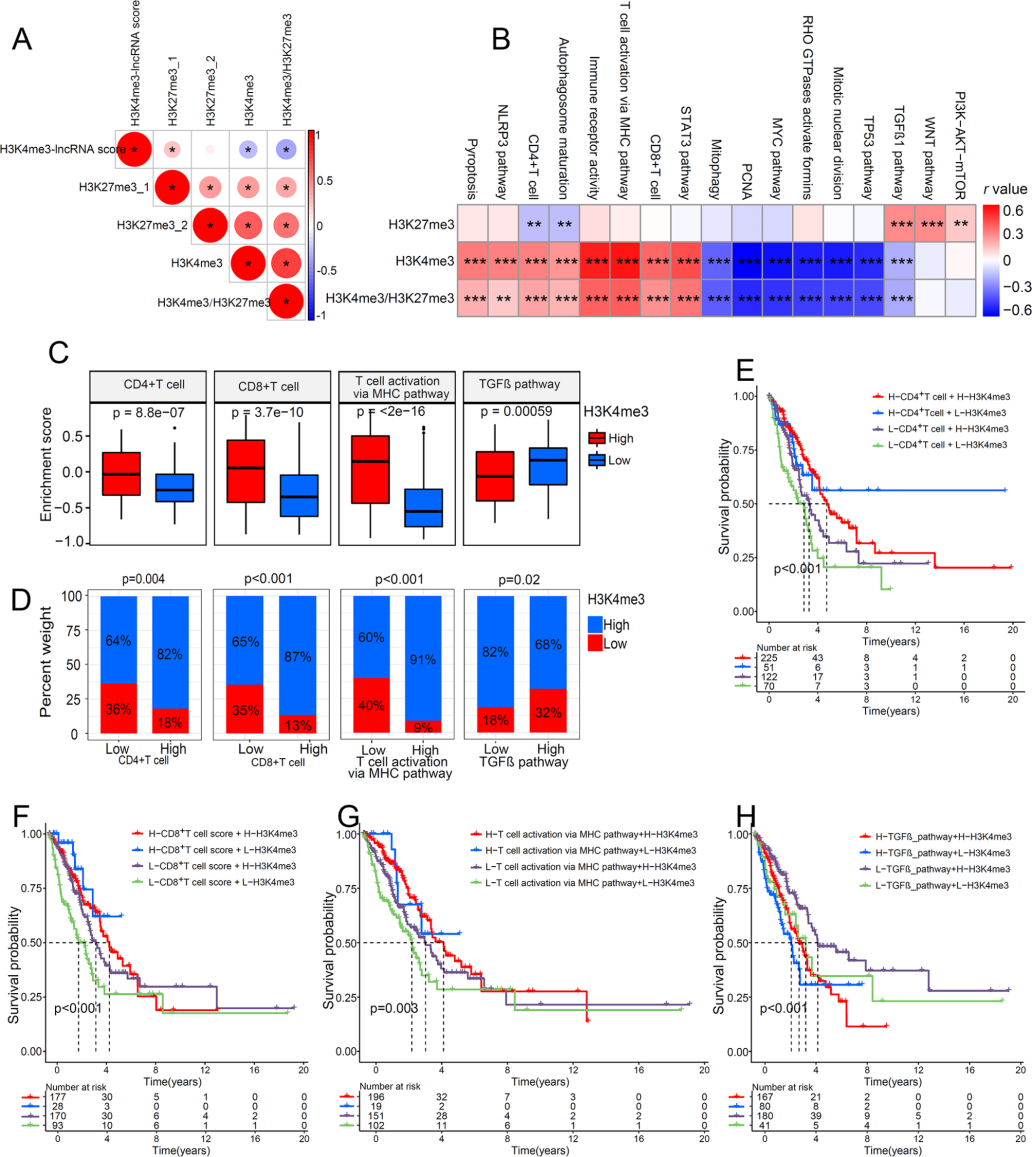
The impact of H3K4me3 on biological process in LUAD. (A) Correlation analysis between H3K4me3-lncRNA score and different histone methylation enrichment scores. (B) The correlation matrix showed the correlation between histone trimethylation and tumor related biological process.(C) Comparison of the enrichment score of CD4^+^ T cell, CD8^+^ T cell, T cell activation via MHC pathway, and TGFβ between high and low H3K4me3 groups. (D) The proportion of different score groups of indicated molecular subtypes in the two H3K4me3 score patterns. (E) (F) (G) (H) Kaplan–Meier curves showing the predictive value of indicated molecular subtypes. E, survival curves based on CD4^+^ T cell activation and H3K4me3 level; F, survival curves based on CD8^+^ T cell activation and H3K4me3 level; G, survival curves based on T cell activation via MHC pathway score and H3K4me3 level; H, survival curves based on TGFβ activation and H3K4me3 level. (*p < 0.05; **p < 0.01; ***p < 0.001)

In order to directly demonstrate the role of H3K4me3 modification in LUAD, we included the H3K4me3 score. We first analysis the H3K4me3 score and other histone methylation modification score. Fig. 5 A showed that the H3K4me3 score was significantly correlated with H3K4me3-lncRNA score. The correlation matrix indicated that the H3K4me3 score was significantly positively correlated with anti-tumor pathways and significantly negatively correlated with pro-tumor pathways (Fig. 5B). Patients with high H3K4me3 score had obvious survival advantages (Fig. S4A). High level of H3K4me3 had low H3K4me3-lncRNA score (Fig. S4B). A Chi-square test revealed a smaller proportion of patients with high H3K4me3 modification in the high-H3K4me3-lncRNA score populations (Fig. S4C). These results indicated that low H3K4me3 score was the initiating factor for poor prognosis of LUAD. Then, we further explored the molecular biological mechanism of low H3K4me3 modification to promote the progression of LUAD. Fig. 5 C showed that patients with a high level of H3K4me3 had increased CD4^+^T cell and CD8^+^T cell activation, and inhibited TGFβ-mediated EMT. ILs- and chemokine- mediated immune pathways were also activated in patients with high H3K4me3 score (Fig. S5A). Interestingly, autophagy-, ferroptosis-, and pyroptosis- mediated programmed cell death were also activated in patients with high level of H3K4me3 (Fig. S5B). At the same time, patients with high H3K4me3 score showed significant suppression of cell proliferation and other classic pro-tumor pathways (Fig. S5C). In addition, A Chi-square test revealed that there was a more significant proportion of patients with activated CD4^+^T cell, CD8^+^T cell, or T cell activation via MHC pathway in the high H3K4me3 populations (Fig. 5D). On the contrary, there was a more significant proportion of patients with inhibited TGFβ-mediated EMT in the high H3K4me3 populations (Fig. 5D). Fig. 5E showed that patients with the high level of H3K4me3 and high CD4^+^T enrichment score had the best survival advantage. Similar results were obtained in Fig. 5F and 5G. However, Fig. 5H showed that patients with a high level of H3K4me3 and low TGFβ enrichment score had the best survival advantage. The survival analysis confirmed that high H3K4me3 modification significantly improve the patients’ survival benefits from activated CD4^+^T cells and CD8^+^T cells.

### The effect of H3K4me3 modification on immune checkpoints (ICs)

**Fig. 6:**
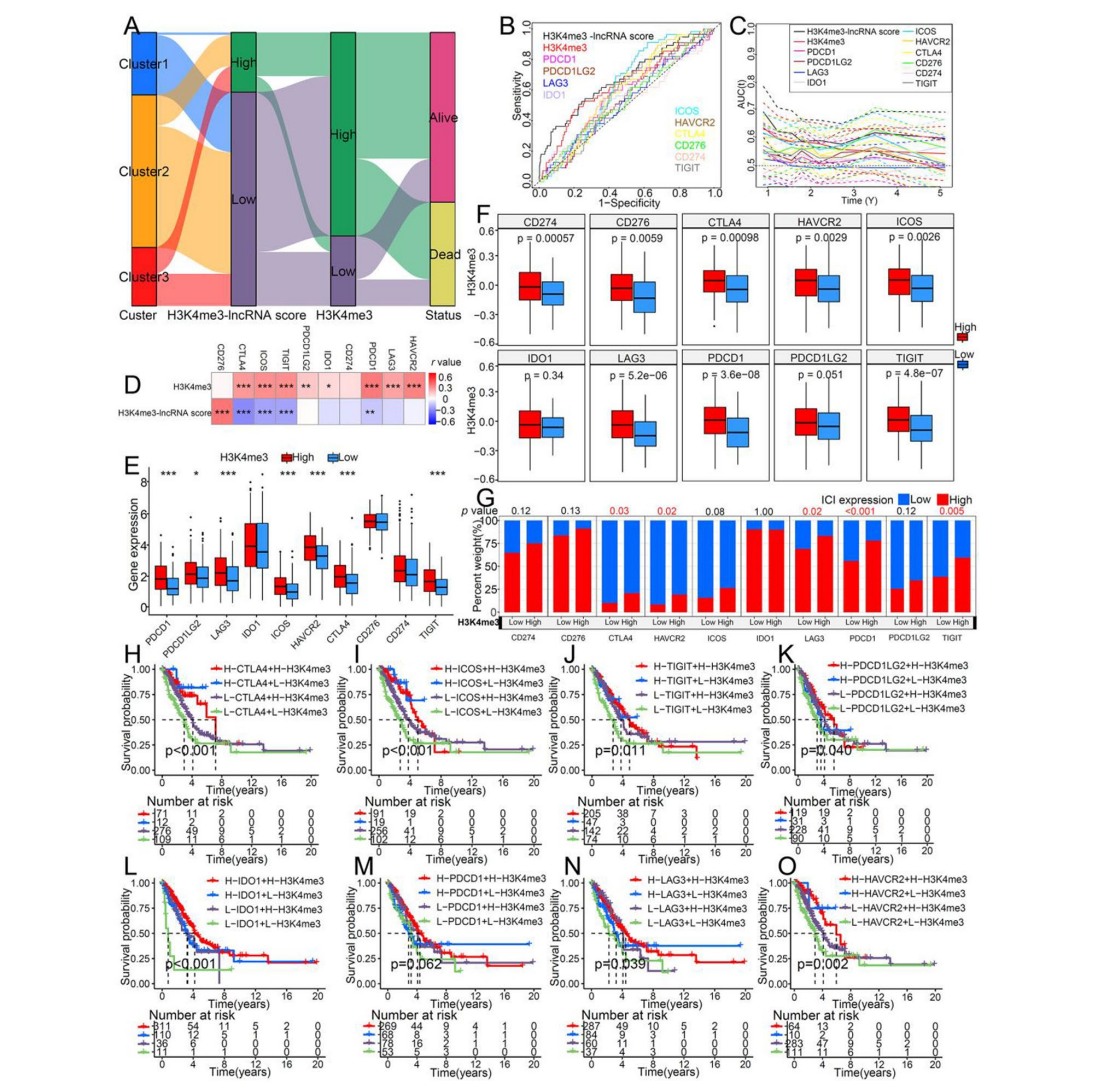
The impact of H3K4me3 modification on immune checkpoint. (A) Alluvial diagram showing the changes of H3K4me3-lncRNA cluster, H3K4me3-lncRNA score and H3K4me3 score. (B) ROC curves of H3K4me3-lncRNA score and immune checkpoints for predicting survival of LUAD in TCGA dataset. (C) Time-dependent AUC of H3K4me3-lncRNA score and immune checkpoints for predicting 1to5-year survival. (D) The correlation matrix showed the correlation between H3K4me3 score and H3K4me3-lncRNA score and immune checkpoints. (E) Comparison of expression of immune checkpoints between high and low H3K4me3 groups. (F) Differences in H3K4me3 between high and low immune checkpoint groups. (G) The proportion of patients with high and low immune checkpoint in the two H3K4me3-lncRNA score types. (H) (I) (J) (K) (L) (M) (N) (O) Kaplan-Meier curves showing the predictive value of indicated H3K4me3 and immune checkpoint subtypes. H, survival curves based on CTLA4 expression and H3K4me3 level; I, survival curves based on ICOS expression and H3K4me3 level; J, survival curves based on TIGIT expression and H3K4me3 level; K, survival curves based on PDCD1LG2 expression and H3K4me3 level; L, survival curves based on IDO1 expression and H3K4me3 level; M, survival curves based on PDCD1 expression and H3K4me3 level; N, survival curves based on LAG3 expression and H3K4me3 level; O, survival curves based on HAVCR2 expression and H3K4me3 level. (*p < 0.05; **p < 0.01; ***p < 0.001)

The changes of the H3K4me3-lncRNA cluster, H3K4me3-lncRNA score and H3K4me3 modification were visualized using an alluvial diagram Fig. 6A. The expression level of ICs is a crucial factor for patients to benefit from immunotherapy. In our study, patients with high expression of ICs showed a survival advantage (Fig. S6A-S6J). Using ROC curves, we evaluated the predictive power of H3K4me3-lncRNA score, H3K4me3 modification, and ICs expression on the prognosis of patients with LUAD (Fig. 6B). The time-dependent AUC indicated that H3K4me3-lncRNA score and H3K4me3 score had a better ability to predict the prognosis of patients (Fig. 6C). Since H3K4me3 can regulate CD4^+^T cell and CD8^+^T cell activation, we speculate that H3K4me3 also plays an vital role in regulating the expression of ICs. The correlation matrix showed that H3K4me3 score was strongly positively correlated with most ICs (Fig. 6D). The expression of PDCD1, PDCD1LG2, LAG3, ICOS, HAVCR2, CTLA4, CD274, and TIGIT in patients with high H3K4me3 score was significantly increased (Fig. 6E). In addition, we found that the H3K4me3 modification was significantly restrained in patients with low expression of ICs (Fig. 6F). A Chi-square test revealed that the proportion of patients with high CTLA4, HAVCR2, LAG2, PDCD1, or TIGIT expression was also higher in people with high H3K4me3 score (Fig. 6G). These results indicated that H3K4me3 modification is closely related to the expression of ICs. Then, as a new classification method, we speculated whether H3K4me3 modification could be combined with ICs to predict the prognosis of LUAD more accurately. The results showed that patients with a high level of H3K4me3 and high expression of CTLA4 (Fig. 6H), ICOS (Fig. 6I), TIGIT (Fig. 6J), PDCD1LG2 (Fig. 6K), IDO1 (Fig. 6L), LAG3 (Fig. 6N), or HAVCR2 (Fig. 6O) had the best survival advantage. Although the survival advantage of patients with high H3K4me3 and high PDCD1 expression was not significant, these patients still had a trend of higher median survival (Fig. 6M). These survival analysis told us that a new treatment method combining immune checkpoint inhibitors (ICIs) with epigenetic modification drugs that promote H3K4me3 might improve the effectiveness of patient immunotherapy.

### H3K4me3 modification in the role of anti-PD-1/anti-PD-L1 immunotherapy

**Fig. 7:**
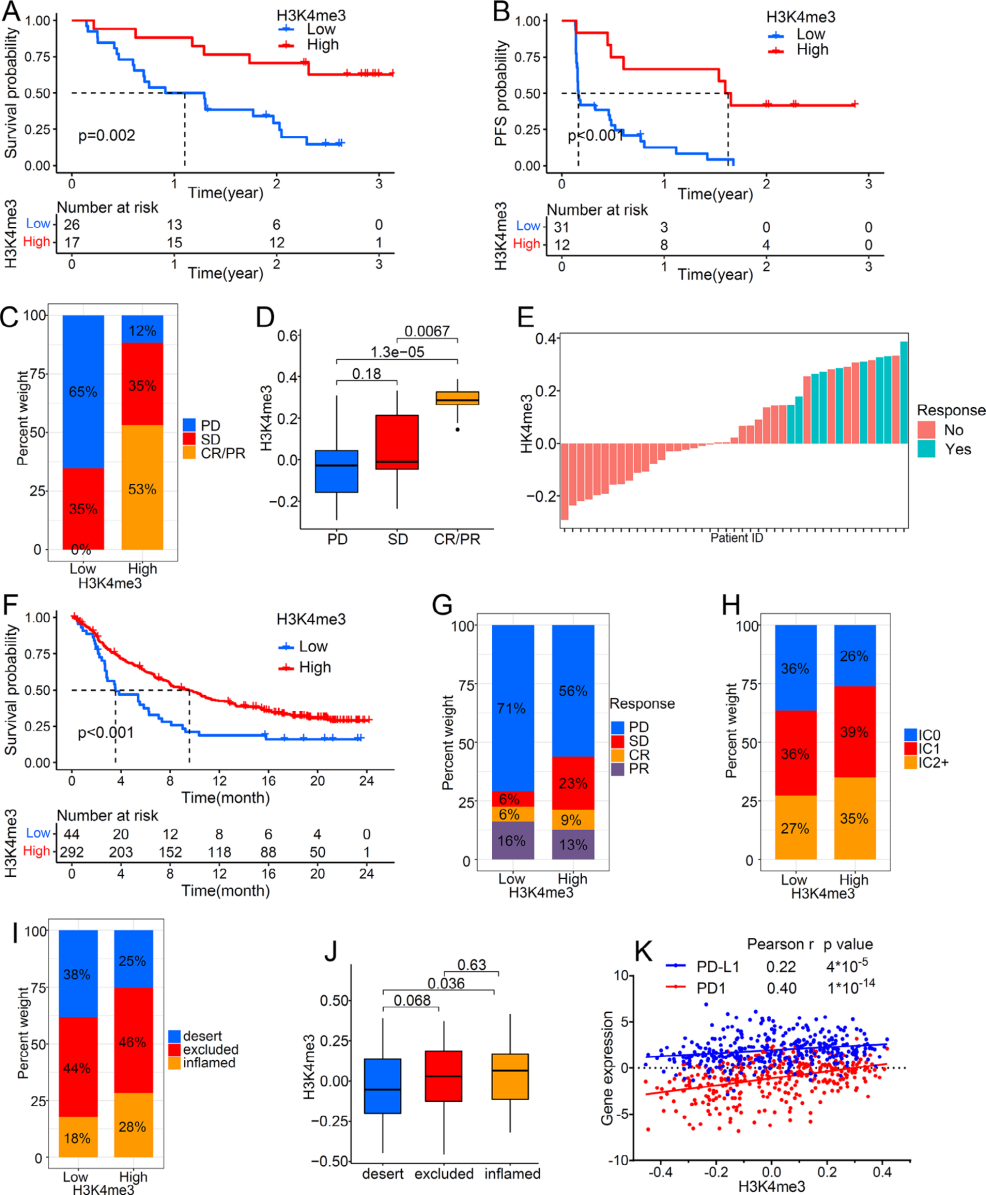
The impact of H3K4me3 modification on anti-PD-1/anti-PD-L1 immunotherapy. (A) OS and PFS (B) analysis for high and low H3K4me3 score based on 43 melanoma patients treated with Nivolumab (anti-PD-1 anti-body). (C) The proportion of patients with response to Nivolumab in high and low H3K4me3 groups. (D) H3K4me3 score in different Nivolumab response groups. (E) The correlation of H3K4me3 with response to Nivolumab. (F) OS analysis for high and low H3K4me3 score based on 336 locally advanced or metastatic urothelial cancer patients treated with Atezolizumab (anti-PD-L1 anti-body). (G) The proportion of patients with response to Atezolizumab in high and low H3K4me3 groups. (H) The proportion of patients with distinct tumor-infiltrating immune cells (IC) in high and low H3K4me3 groups. (I) The proportion of patients with distinct tumor immune phenotypes in high and low H3K4me3 groups. (J) H3K4me3 score in different tumor immune phenotypes. (K) Correlation analysis between H3K4me3 and PD-1/PD-L1

Considering the close relationship between H3K4me3 and immune checkpoints, we further explored the role of H3K4me3 modification in the treatment of ICIs. Melanoma patients (GSE91061 cohort) received Nivolumab treatment (anti-PD-1 anti-body) with high H3K4me3 score had significant overall survival (OS) and progression-free survival (PFS) advantages (Fig. 7A, B). Patients with high H3K4me3 score had significant therapeutic advantages (Fig. 7C, D) and clinical response (Fig. 7E) to anti-PD-1. In order to better illustrate the impact of H3K4me3 on the effect of immunotherapy, an another large-sample anti-PD-L1 cohort (IMvigor210) was included. As expected, Patients with high H3K4me3 score receiving anti-PD-L1 therapy showed a significant survival advantage (Fig. 7F). Similarly, in the low H3K4me3 group, the proportion of patients with disease progression was higher (Fig. 7G). PD-L1 expression on tumor-infiltrating immune cells (IC) was associated with tumor immunotherapy response [26]. IC2/3 group had a significant survival advantage [26]. In the high H3K4me3 group, the proportion of IC2 + patients was increased (Fig. 7H). We next investigated the relationship between H3K4me3 and immune status to clarify the reason for H3K4me3 improving immunotherapy response. The result indicated that a high H3K4me3 score was remarkably associated with inflamed immune phenotypes (Fig. 7I, J). In addition, we found that H3K4me3 score has a significant positive correlation with PD-1 and PD-L1 (Fig. 7K). Our results showed that H3K4me3 modification is irreplaceable in response to anti-PD-1/L1 immunotherapy and tumor microenvironment remodeling.

### Immunohistochemical validation of the role of H3K4me3 in LUAD

**Fig. 8:**
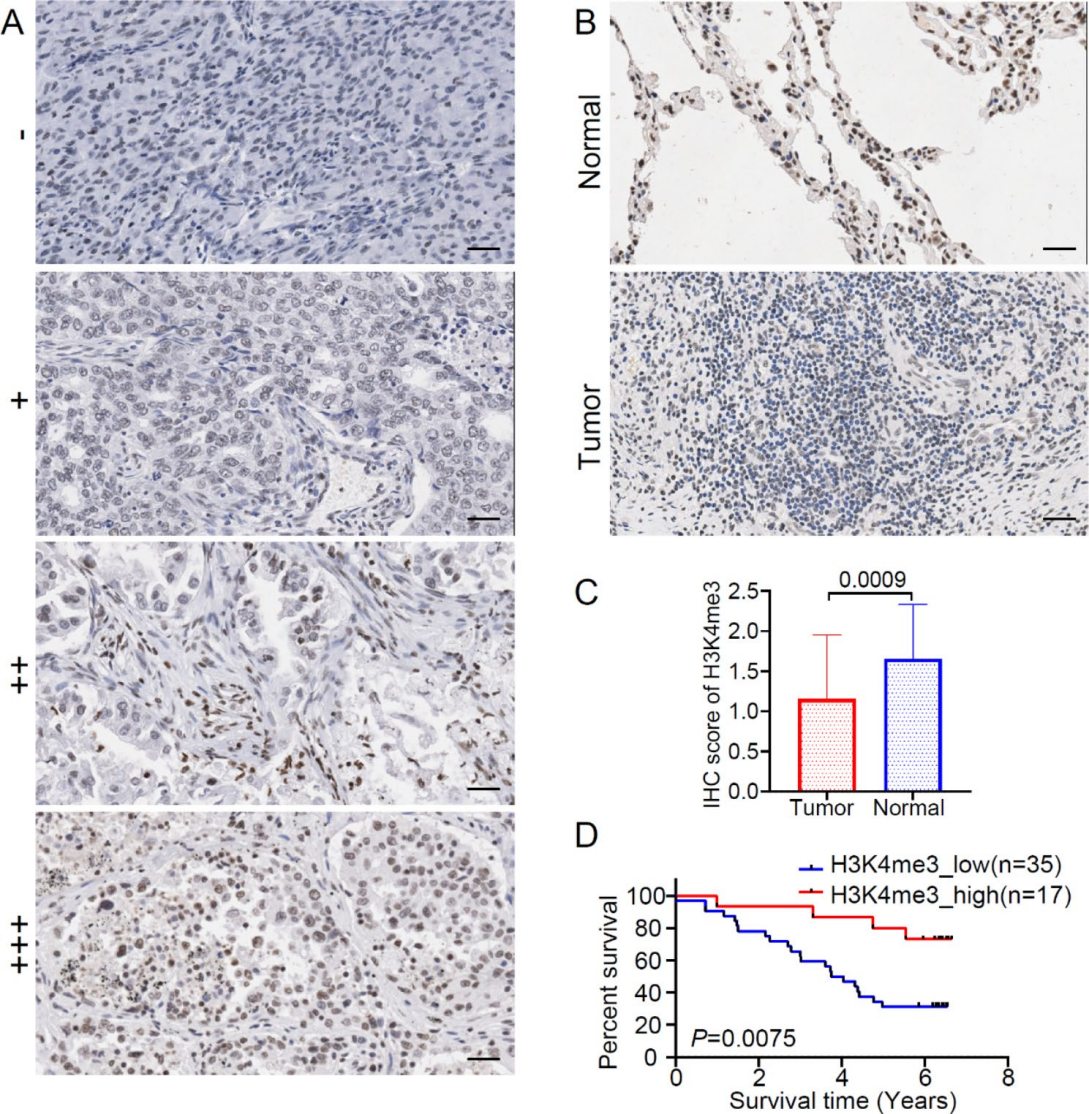
Protein expression level of H3K4me3 in LUAD and its effect on the prognosis of patients. (A) The expression of H3K4me3 was divided into 4 levels: “-” (negative), “++” (low), “++” (moderate) or “+++” (high). (B) Immunohistochemical staining showed H3K4me3 protein expression in LUAD tumor tissue and their adjacent normal tissue. (C) Comparison of H3K4me3 expression levels in tumor tissue and paracancerous tissue. (D) OS analysis for high and low H3K4me3 protein levels based on 52 LUAD patients. (Scale bar, 100 μm)

Although we have previously proved that patients with high H3K4me3 had a good prognosis due to the H3K4me3-mediated enhanced immune sensitivity, the H3K4me3 level was indirectly evaluated by a gene set enrichment score [27]. Here, we collected a total of 52 LUAD patients as independent validation. The staining intensity was divided into 4 levels: “-” (negative), “+” (low), “++” (moderate) or “+++” (high) (Fig. 8A). Immunohistochemical staining showed H3K4me3 protein expression in LUAD tumor tissues and their adjacent normal tissues (Fig. 8B). The H3K4me3 protein level in tumor tissues was significantly lower than that in adjacent tissues (Fig. 8C). Patients were divided into high and low H3K4me3 groups based on the IHC score. Patients in high H3K4me3 group had significant survival advantage (Fig. 8D). The results showed that high expression of H3K4me3 is a favorable factor for lung cancer patients.

## Discussion

Many studies have shown that epigenetic modifications are involved in tumorigenesis, invasion and metastasis [28–33]. The H3K4me3 modification has long been regarded as one of the most critical epigenetic modifications that regulate gene transcription. H3K4me3 provides a binding site for TFIID to promote transcription activation [2, 3, 34]. However, whether H3K4me3 modification promotes tumors or suppresses tumors is still controversial [5–8]. These disputes may be attributed to the fact that these studies were only based on several cell lines, individual genes, or signaling pathways. The characteristics of the immune microenvironment based on multiple H3K4me3 regulators and their highly correlated lncRNAs have yet to be fully understood. In this study, for the first time, we clarified the characteristics of H3K4me3 modification in LUAD tumor microenvironment based on specific lncRNAs, and found that H3K4me3 modification was closely related to immune cell infiltration, CD4 + T cell and CD8 + T cell activation, EMT, programmed cell death and other tumor-related pathways. Secondly, we have established a prediction model based on the expression of H3K4me3-related lncRNAs, which could provide a better patient enrollment criteria for tumor immunotherapy targeting CD4^+^ or CD8^+^ T cells. In addition, based on the sequencing data of the largest clinical sample size, we found that a low level of H3K4me3 was the initiating factor for the poor prognosis of LUAD. A high level of H3K4me3 modification promoted the activation of CD4^+^ and CD8^+^ T cells, induced programmed cell death, and inhibited cell proliferation and TGFβ-mediated EMT. More importantly, we comprehensively analyzed the relationship between H3K4me3 modification and ICs, and we verified that high H3K4me3 level significantly enhanced the clinical therapeutic effect of anti-PD-1/anti-PD-L1 antibody based on two immunotherapy cohorts. Meanwhile, the clinical specimens collected from 52 patients with LUAD also proved once again the beneficial effect of high H3K4 expression on the long-term survival of patients.

The activation state of T cells played a decisive role in the effect of tumor immunotherapy. As reported in the literature, activated CD4^+^ T cells maintained the activation of NK cells and contributed to sustained antibody-dependent cellular cytotoxicity [35]. In addition, asparagine potentiated CD8^+^ T cell activation to promote anti-tumor effects [36]. Our data indicated that patients with high H3K4me3 had increased CD4^+^T cell and CD8^+^T cell activation. Consistent with the literature, our study confirmed that patients with high activation of CD4 + T cell or CD8 + T cell had obviously survival advantages. Considering that patients with potentiated CD4 + T cell or CD8 + T cell activation had increased H3K4me3 modification and that patients with high H3K4me3 level and T cell activation had the most prolonged median survival, we speculated that high H3K4me3 modification could significantly improve the patient’s survival benefits from activated CD4^+^T cell and CD8^+^T cell. As a result, increasing H3K4me3 modification combined with drugs targeting CD4^+^ or CD8^+^ activation may enable patients to benefit more from immunotherapy.

ICI therapy has been the most crucial part of cancer treatment in recent years. Although the therapeutic effect of ICIs is not good enough, the survival benefits it brings to patients are irreplaceable. Identifying specific populations suitable for ICI therapy to achieve individualized treatment and discovering novel drugs combined with ICIs to improve therapeutic effects are tumor immunotherapy’s two most important links and goals. Based on this consideration, we first constructed an H3K4me3-lncRNA score signature, which showed substantial power in predicting the prognosis of LUAD patients compared with other biomarkers. Second, we assessed the H3K4me3 level of each sample and found that patients with high level of H3K4me3 had a significant survival advantage. Meaningfully, H3K4me3 was strong positively correlated with CTLA4, ICOS TIGIT, PDCD1LG2, IDO1, CD274, PDCD1, LAG3, and HAVCR2, and these ICs were significantly over-expressed in patients with high H3K4me3 levels. Survival analysis indicated that patients with high level of H3K4me3 and high expression of ICs had the best survival advantage, which brought us a new treatment method combining ICIs with epigenetic modification drugs that promote H3K4me3 may improve the effectiveness of patient immunotherapy.

## Conclusions

For the first time, we comprehensively analyzed the expression signature of H3K4me3-related lncRNAs, and clarified the characteristics of H3K4me3 modification in the TME of LUAD. Based on the maximum sample size, we discovered the positive effects of H3K4me3 on the prognosis of LUAD and explored the immunological mechanism of H3K4me3 for tumor suppression. Meaningfully, high expression of H3K4me3 could significantly enhance the clinical treatment response of anti-PD-1/anti-PD-L1 antibody. These results indicated that a new treatment method combining ICIs with epigenetic modification drugs that promote H3K4me3 might allow patients to benefit more from immunotherapy.

## Electronic supplementary material

Below is the link to the electronic supplementary material.

Supplementary Material 1

Supplementary Material 2

Supplementary Material 3

Supplementary Material 4

Supplementary Material 5

Supplementary Material 6

Supplementary Material 7

## Data Availability

The analyzed data in this study are available from the corresponding author on reasonable request. The raw data used in the supplement figures was showed in Table S6.

## List of abbreviations

H3K4me3: Trimethylation of lysine 4 on histone H3 protein subunit
LUAD: Lung adenocarcinoma
LncRNA: Long non-coding RNA
CTLA4: Cytotoxic T-lymphocyte-associated protein 4
ICOS: Inducible T cell costimulatory
TIGIT: T cell immunoreceptor with Ig and ITIM domains
PDCD1LG2: Programmed cell death 1 ligand 2
IDO1: Indoleamine 2,3-dioxygenase 1
PDCD1: Programmed cell death 1
LAG3: Lymphocyte activating 3
HAVCR2: Hepatitis A virus cellular receptor 2
TME: Tumor microenvironmentDEGs:Differentially expressed genes
GSVA: Gene set variation analysis
EMT: Epithelial-mesenchymal transition
ICs: Immune checkpointsIC:Tumor-infiltrating immune cells
TME: Tumor microenvironment
ICIs: Immune checkpoint inhibitors
OS: Overall survival
PFS: Progression-free survival
GO: Gene ontology
KEGG: Kyoto encyclopedia of genes and genomes
GSEA: Gene set enrichment analysis
FDR: false discovery rate
PCA: Principal component analysis
AUC: Area under the curve
