# Supplementary material for: H3K4me3-related lncRNAs signature and comprehensive analysis of H3K4me3 regulating tumor immunity in lung adenocarcinoma
Source: Respir Res. 2023 May 3;24:122. doi: 10.1186/s12931-023-02418-1 (PMC10155324; doi:10.1186/s12931-023-02418-1)
Supplement: Supplementary file 1 — Supplementary Material 1 [file 12931_2023_2418_MOESM1_ESM.doc]

**Fig. S1** Flowchart of this work.

**Fig. S2** Expression characteristics of lncRNA closely related to H3K4me3 in LUAD. (A) The network of lncRNAs and H3K4me3 regulators. (B) Univariate analysis identified H3K4me3-related prognostic lncRNAs. (C) The expression difference characteristics of identified lncRNAs between normal and tumor tissues.


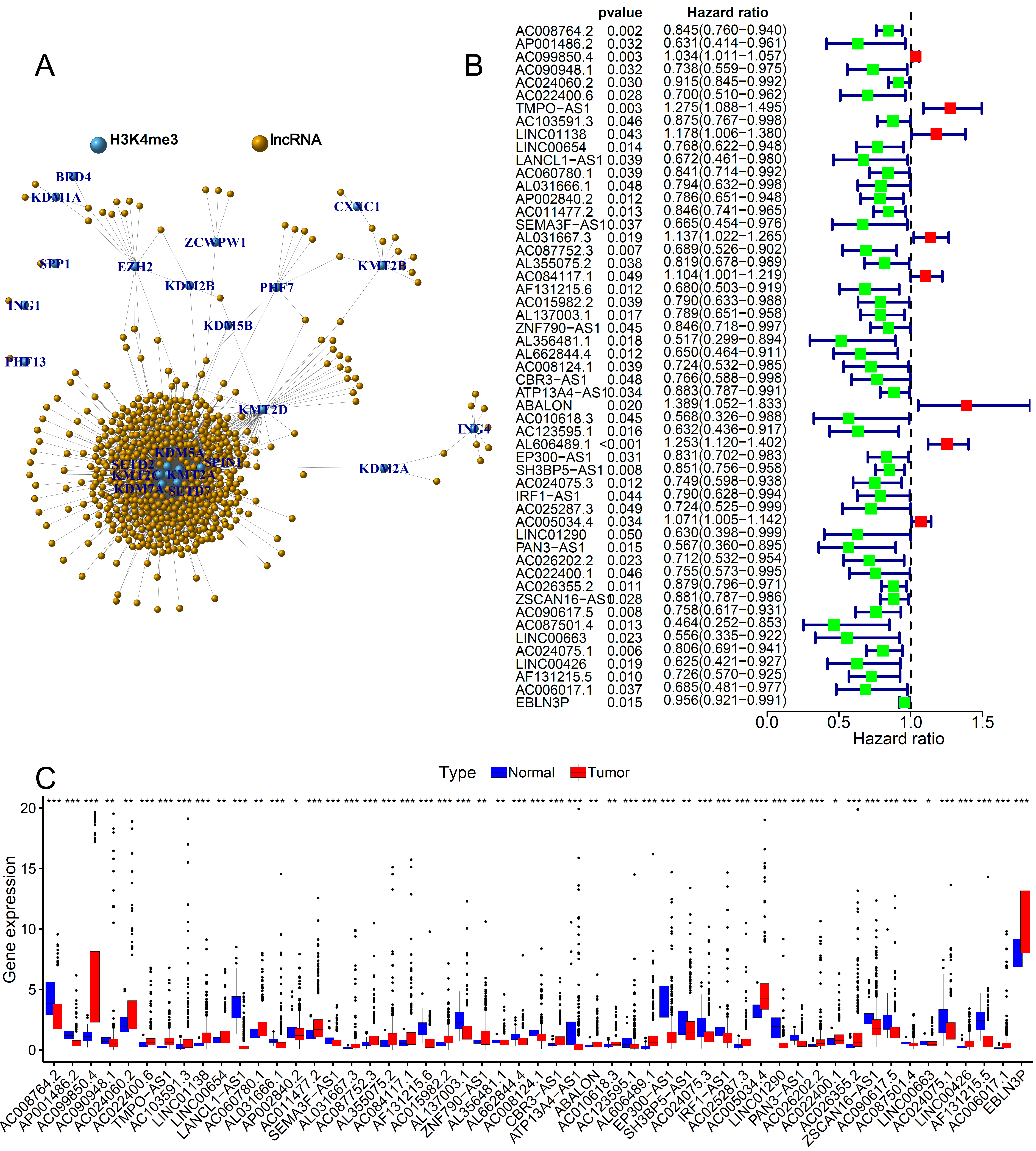


**Fig. S3** (A) (B) GO and KEGG analysis for differently expressed genes between high and low H3K4me3-lncRNA score. (C) GSEA_HALLMARK term analysis for high and low H3K4me3-lncRNA score (adj. *p*<0.05; *p* value<0.05; NES, enrichment score). (D) GSEA_GO term analysis for high and low H3K4me3-lncRNA score (adj. *p*<0.05; *p* value<0.05; NES, enrichment score; The top 10 positive and negative pathways selected by NES were provided).


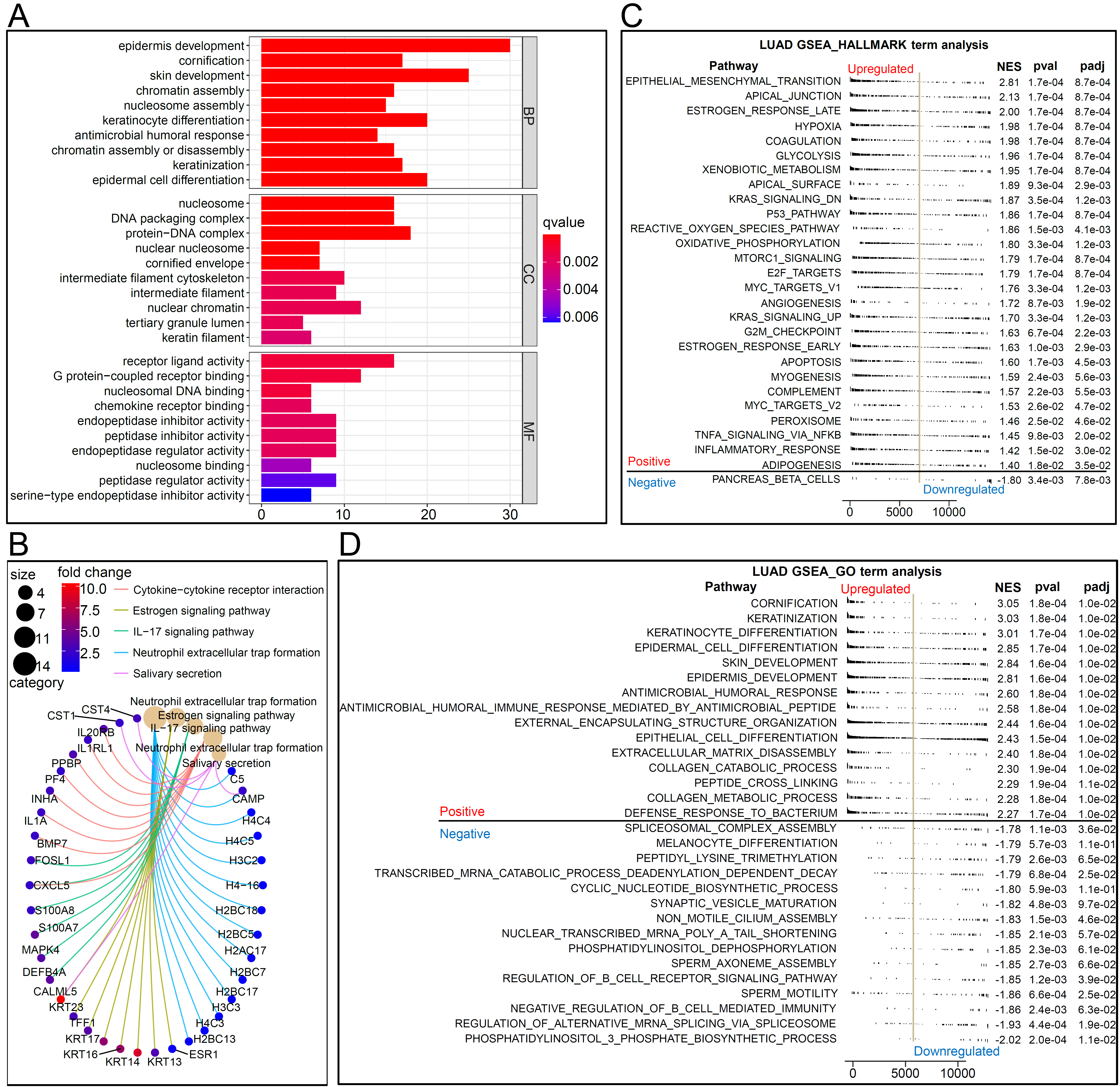


**
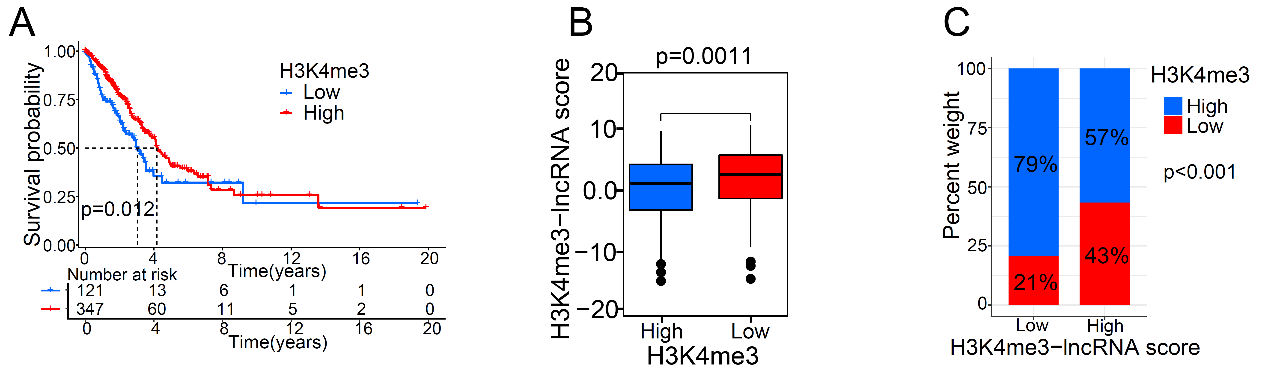
**

**Fig. S4** The effect of H3K4me3 on the prognosis of patients with LUAD and its relationship with H3K4me3-lncRNA score. (A) Survival analyses showed a significant impact of H3K4me3 on outcome. (B) Differences in H3K4me3-lncRNA score between high and low H3K4me3 level. (C) The proportion of patients with high and low H3K4me3 level in the two H3K4me3-lncRNA score types.

**Fig. S5** Comparison analysis of tumor-related biological process between patients with high and low H3K4me3. (A) Comparison analysis of immune cell activation and ILs- and chemokine- mediated pathways between patients with high and low H3K4me3. (B) Comparison analysis of programed cell death between patients with high and low H3K4me3. (C) Comparison analysis of cell repair, cell proliferation, EMT, and other pro-tumor pathways between patients with high and low H3K4me3.


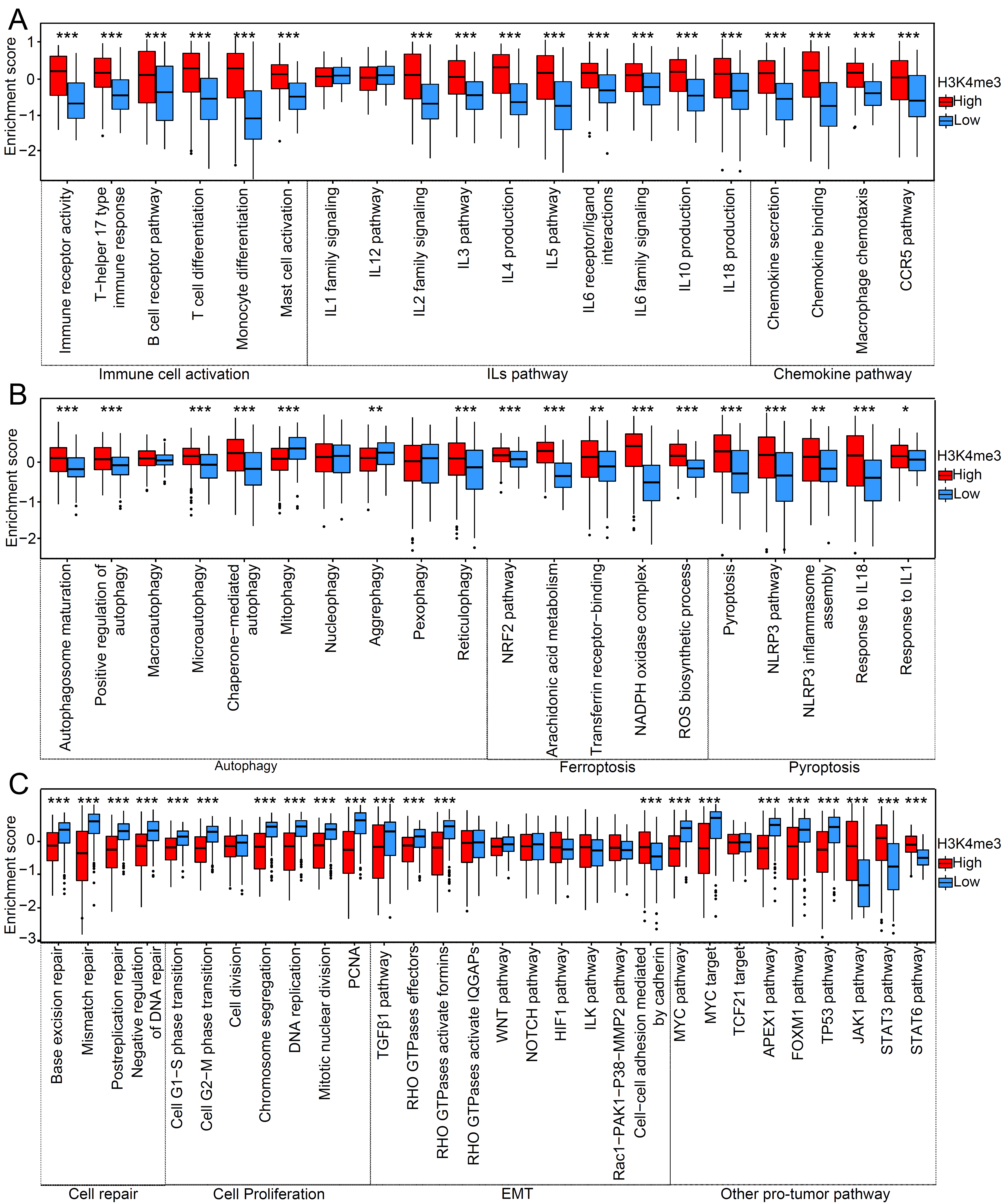

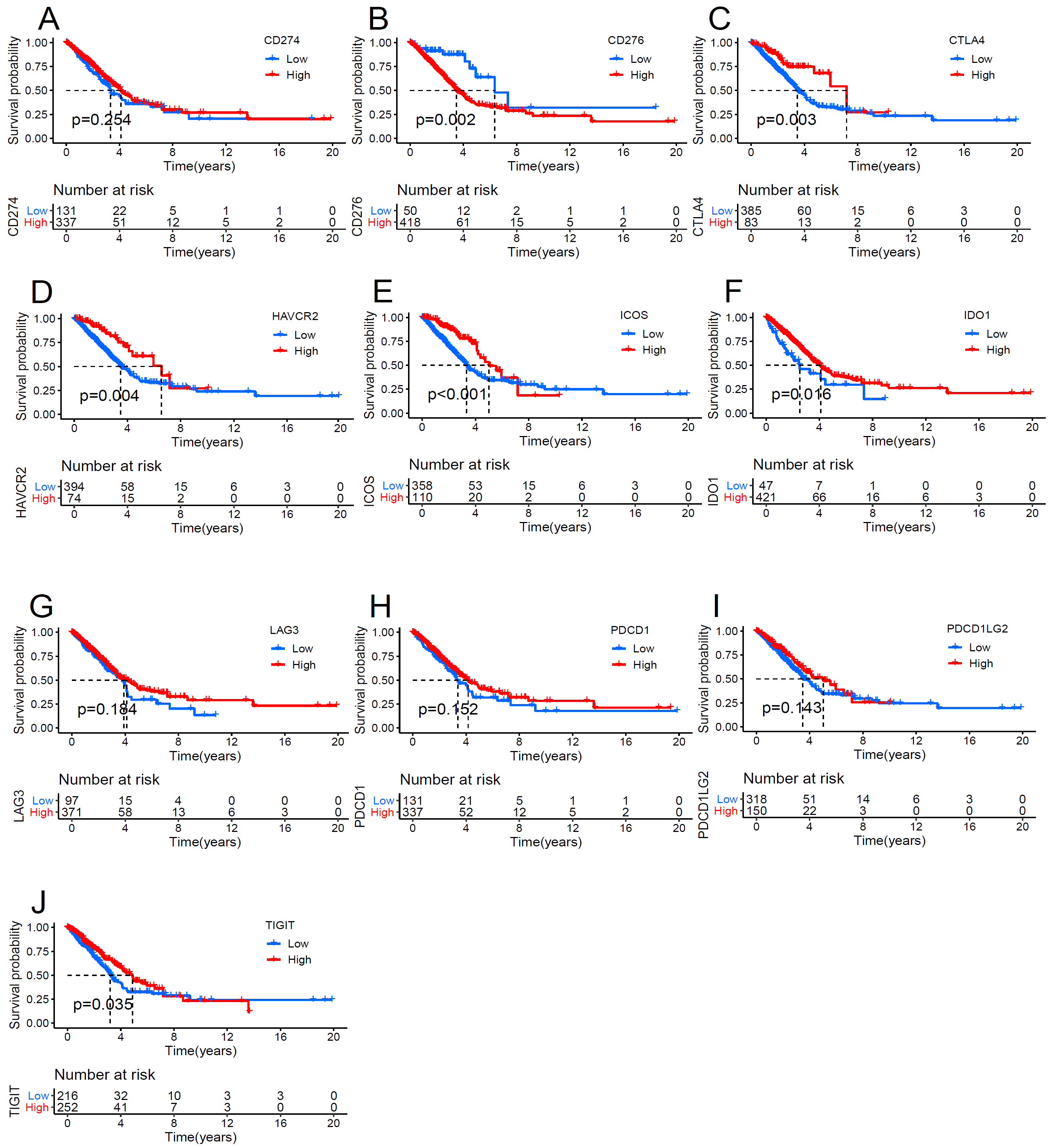


**Fig. S6** Overall survival analysis for the indicated subgroups. Kaplan–Meier curves showing the impact of the expression of CD274(A), CD276(B), CTLA4(C), HAVCR2(D), ICOS(E), IDO1(F), LAG3(G), PDCD1(H), PDCD1LG2(I), and TIGIT(J) on patients’ survival.
